# Digital Rectal Examination in the Imaging Era: Not Abandoned, but Repositioned

**DOI:** 10.1590/S1677-5538.IBJU.2026.0508

**Published:** 2026-07-30

**Authors:** Anil Eker, Serdar Çelik

**Affiliations:** 1 Izmir City Hospital Urology Clinic Izmir Turkey Urology Clinic, Izmir City Hospital, Bayrakli, Izmir, Turkey; 2 University of Health Sciences Izmir Faculty of Medicine Department of Urology Izmir Turkey Department of Urology, University of Health Sciences, Izmir Faculty of Medicine, Bayrakli, Izmir, Turkey

To the editor,

We read with great interest the expert opinion by Reis, entitled "Repositioning the Digital Rectal Examination in the Era of Risk-Adapted Prostate Cancer Diagnostics: Right Tool to the Right Patient at the Right Time," published in the July–August 2026 issue of the International Brazilian Journal of Urology. The author provides a timely and balanced perspective on the evolving role of digital rectal examination (DRE) in contemporary prostate cancer diagnostics (1).

We fully agree with the central message of the article: DRE should no longer be regarded as a frontline population-based screening tool, but it should not be considered obsolete. Rather, its current value lies in selected clinical scenarios, particularly when interpreted within a risk-adapted and multimodal diagnostic pathway. As highlighted by the author, DRE has important limitations, including low sensitivity, operator dependence, and limited ability to detect anterior or small-volume tumors. These limitations have become even more apparent with the expanding role of mpMRI, which improves the detection of clinically significant prostate cancer and the prediction of pathological grade. Accordingly, DRE has increasingly been relegated to a secondary role in contemporary diagnostic pathways (2). Nevertheless, a clearly suspicious DRE may still provide meaningful clinical information, especially when imaging findings are equivocal or discordant with clinical suspicion (3).

In line with this perspective, recent studies have increasingly explored the relationship between DRE findings and modern imaging parameters, including mpMRI-derived PI-RADS scores, lesion size, and PSMA PET/CT uptake (4, 5). Our recent clinical data also support this concept. In a cohort of 164 patients with prostate cancer, suspicious DRE findings were associated with higher PI-RADS scores, larger mpMRI lesion size, elevated PSMA PET/CT SUVmax, clinically significant prostate cancer, and advanced pathological stage. Notably, each one-point increase in PI-RADS score was associated with a 2.14-fold increase in the likelihood of suspicious DRE findings. We also identified clinically relevant thresholds for DRE positivity, including an mpMRI lesion size of 13.5 mm and a PSMA PET/CT SUVmax of 15.1. These observations suggest that DRE positivity may reflect not only anatomical palpability, but also tumor burden and biological aggressiveness (5).

Therefore, the contemporary role of DRE should be defined more precisely. It is not a replacement for PSA, mpMRI, or PSMA PET/CT, nor should it be used as a stand-alone screening tool. However, when abnormal, DRE may serve as a simple, low-cost, and immediately available clinical marker that strengthens suspicion for high-volume or advanced disease. It should be acknowledged that the intimate nature and manner of performing DRE may cause additional stress, while investigation for possible cancer and the prospect of a cancer diagnosis may themselves provoke anxiety and depression. Therefore, the examination should be conducted within a patient-centered framework, with clear communication about its purpose, benefits, and limitations (6).

We congratulate the author for emphasizing this important distinction. The future of prostate cancer diagnostics should not rely on replacing traditional clinical examination with imaging alone, but on integrating clinical judgment, biomarkers, and advanced imaging to identify the right patient at the right time.

The Authors
